# A Review of Pseudomyxoma Peritonei: Insights Into Diagnosis, Management, and Prognosis

**DOI:** 10.7759/cureus.61244

**Published:** 2024-05-28

**Authors:** Siddhi Shringi, Anil K Agrawal, Pravin Gadkari

**Affiliations:** 1 Pathology, Jawaharlal Nehru Medical College, Datta Meghe Institute of Higher Education and Research, Wardha, IND

**Keywords:** prognosis, perioperative intraperitoneal chemotherapy, cytoreductive surgery, management, diagnosis, pseudomyxoma peritonei

## Abstract

Pseudomyxoma peritonei (PMP) is a rare and complex clinical syndrome characterized by the accumulation of mucinous ascites within the peritoneal cavity, typically associated with mucinous tumours of appendiceal origin. Despite its rarity, PMP poses significant challenges in diagnosis and management due to its indolent yet locally aggressive nature. This comprehensive review provides insights into the diagnosis, management, and prognosis of PMP, synthesizing current evidence and emerging trends in the field. Challenges and opportunities in PMP management are discussed, along with recommendations for clinical practice emphasizing the importance of a multidisciplinary approach and specialized care. Despite ongoing challenges, advances in surgical techniques, perioperative chemotherapy, and emerging therapies offer hope for improved outcomes and quality of life for PMP patients.

## Introduction and background

Pseudomyxoma peritonei (PMP) is a rare and complex clinical syndrome characterized by the accumulation of mucinous ascites within the peritoneal cavity. This condition is typically associated with the presence of mucinous tumours, most commonly originating from the appendix, although it can arise from other gastrointestinal organs as well. PMP is known for its indolent yet locally aggressive behaviour, leading to significant morbidity and mortality if left untreated [1]. The history of PMP dates back to the late 19th century when it was first described by Rokitansky in 1842. However, in the 20th century, Dr. Paul K. Sugarbaker pioneered extensive research and treatment strategies for this condition. His work laid the foundation for modern approaches to managing PMP, mainly through cytoreductive surgery (CRS) combined with hyperthermic intraperitoneal chemotherapy (HIPEC) [2].

PMP is considered an exceedingly rare disease, with an estimated annual incidence of less than one to four per million individuals. While it can affect individuals of any age, it most commonly presents in adults aged 50 to 60 years. The incidence of PMP is increasing, possibly due to improved diagnostic techniques and heightened awareness among clinicians [3]. Early and accurate diagnosis of PMP is paramount due to its potential for extensive peritoneal spread and significant morbidity. Furthermore, effective management strategies, including optimal surgical techniques and perioperative chemotherapy, can significantly impact patient outcomes and quality of life. However, PMP poses numerous diagnostic and therapeutic challenges, necessitating a multidisciplinary approach for successful management [4]. This comprehensive review aims to provide insights into the diagnosis, management, and prognosis of PMP. This review seeks to offer guidance for clinicians caring for PMP patients and stimulate further research and innovation in this field by synthesizing current knowledge and emerging evidence.

## Review

Clinical presentation

Signs and Symptoms

PMP typically manifests with a spectrum of symptoms, among which abdominal or pelvic pain stands out as a prevalent complaint among patients. Additionally, individuals often notice a gradual increase in waist circumference, a notable indicator of the disease. Bloating, stemming from the accumulation of mucin within the abdominal cavity, is another frequently reported symptom [5]. Digestive disturbances, such as alterations in bowel patterns, are also commonplace, as PMP can hinder digestion processes and elicit discomfort. In certain instances, PMP may precipitate hernias due to the pressure exerted by mucin against the abdominal wall. As the disease advances, patients may experience diminished appetite due to the discomfort and pain associated with the condition. Abdominal distension, colloquially termed the "jelly belly", represents a prominent symptom of PMP, while abdominal pain can span from mild to severe. Irritation and erythema in the abdominal region are documented symptoms, alongside dyspepsia resulting from organ compression and impeded digestion triggered by mucin accumulation. These symptoms exhibit variability in severity and may not invariably manifest in every patient, occasionally leading to an incidental diagnosis during imaging investigations or surgical interventions for unrelated medical issues [6].

Diagnostic Challenges and Differential Diagnosis

**Figure 1:**
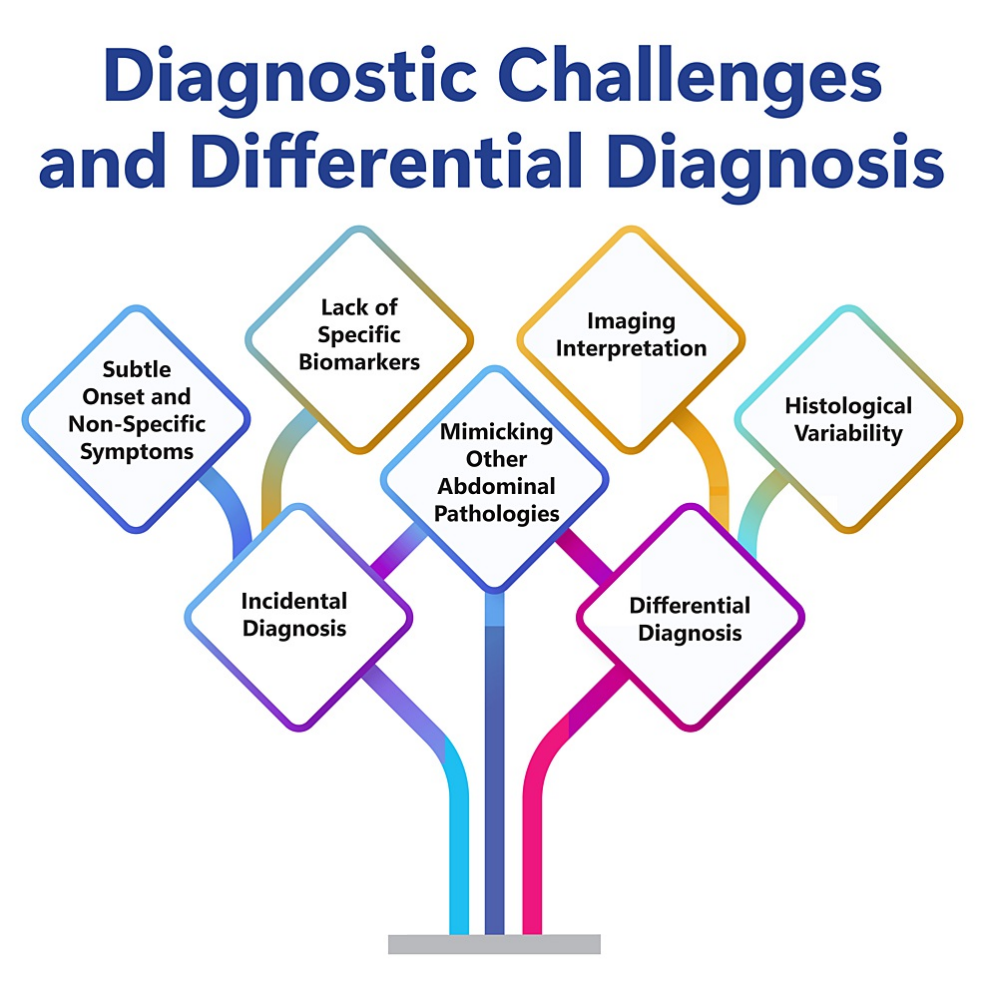
Diagnostic challenges and differential diagnosis. The image is created by the corresponding author.

The diagnostic complexities of PMP stem from its insidious onset and nonspecific symptomatology, frequently posing challenges in accurate identification. Within the realm of differential diagnosis, PMP shares similarities with conditions such as endometriosis exhibiting myxoid alterations, goblet cell carcinoid tumours, and myxoid neoplasms [7]. Clinical manifestations associated with PMP, including abdominal distension, pain, ascites, and intestinal obstruction, often mirror those of other abdominal pathologies, further complicating diagnostic endeavours [8]. Imaging modalities such as ultrasound, computed tomography (CT), or magnetic resonance imaging (MRI) are commonly employed to provide initial insights [7,8] to navigate towards a definitive diagnosis. However, definitive confirmation typically necessitates laparoscopic exploration to obtain histopathological verification of PMP [7]. Moreover, histological examination assumes paramount importance in confirming PMP diagnosis, involving the identification of mucinous cystadenocarcinoma characterized by extracellular and intracellular mucin production [7]. Figure 1 shows diagnostic challenges and differential diagnosis.

Imaging Modalities in PMP Diagnosis

Imaging plays a pivotal role in the diagnostic pathway of PMP, with CT scans with contrast and ultrasonography (US) as primary modalities. Among these, CT scans are deemed the preferred imaging modality for diagnosing PMP, owing to their capacity to discern critical disease features such as peritoneal and omental nodules, visceral scalloping, and non-mucinous ascites [9,10]. Concurrently, US also contributes to PMP diagnosis, albeit its diagnostic efficacy remains subject to ongoing scrutiny [11]. Furthermore, in select instances, endoscopic ultrasound (EUS) has emerged as a diagnostic avenue for PMP [12]. In summation, a synergistic utilization of these imaging techniques, prominently CT scans and US, is indispensable for the accurate diagnosis and comprehensive evaluation of PMP.

Laboratory Findings and Biomarkers

Laboratory findings and biomarkers assume a pivotal role in both the diagnosis and management of PMP. A comprehensive study exploring carbonic anhydrase II (CA II) protein expression among PMP patients emerged as a novel biomarker intricately linked with survival outcomes [13]. Moreover, investigations into circulating serum microRNAs have underscored their potential as diagnostic biomarkers for PMP, emphasizing the significance of molecular markers in the diagnostic landscape of this condition [14]. Additionally, serum tumour markers and histological findings are indispensable prognostic indicators in PMP diagnosis and therapeutic decision-making [8]. These markers, alongside the histological type of neoplasia, are pivotal in guiding treatment strategies and predicting patient outcomes. Notably, the gold standard treatment paradigm for PMP entails complete cytoreductive surgery (CRS), followed by hyperthermic intraperitoneal chemotherapy (HIPEC), with histological diagnosis serving as a cornerstone in determining the optimal therapeutic approach [8].

Diagnostic workup

Clinical Assessment and History-Taking

Clinical assessment and history-taking are paramount in diagnosing and managing PMP. Patients with PMP may present with a constellation of symptoms, including abdominal pain, increased abdominal girth, acute abdomen, or incidental findings during imaging conducted for unrelated conditions [15]. A retrospective analysis underscored abdominal pain as the predominant chief complaint during the initial evaluation of PMP cases, thereby underscoring the critical role of recognizing these symptoms during the history-taking process [15]. Moreover, the study elucidated that suspected ovarian tumours constituted the most frequent indication for surgery in PMP cases, emphasizing the necessity for a comprehensive clinical assessment to delineate PMP from other intra-abdominal pathologies [15]. The study also highlighted the modest accuracy of preoperative diagnostics for PMP, underscoring the diagnostic challenges posed by its mimetic nature. A meticulous clinical assessment, including thorough history-taking to discern symptoms such as abdominal pain, increased abdominal girth, and acute abdomen, emerges as imperative in the prompt detection and judicious management of PMP [16].

Imaging Studies (CT, MRI, and Ultrasound)

Imaging studies serve as cornerstone tools in diagnosing and managing PMP, with CT, MRI, and ultrasound standing as primary modalities. These imaging techniques are instrumental in assessing disease extent, identifying characteristic PMP features, and informing treatment decisions [9,11,17]. CT imaging, in particular, holds significant diagnostic value in delineating the extent of PMP, discriminating it from other peritoneal tumours, and prognosticating tumour grade through specific CT manifestations such as ascites, hepatic scalloping, omental and peritoneal lesion characteristics, intralesional calcification, septa, and peripheral organ involvement [9,17]. Similarly, MRI contributes to PMP evaluation by furnishing detailed peritoneal cavity images and aiding in the differentiation between low-grade and high-grade PMP based on low-grade vs. high-grade MRI features [17]. While the diagnostic utility of ultrasound in PMP is subject to ongoing investigation, it can still play a pivotal role in the initial assessment of patients suspected of PMP, offering a non-invasive imaging alternative that may assist in both diagnosis and disease monitoring [11].

Cytology and Histopathological Examination

Cytology is a diagnostic technique that entails the examination of individual cells or clusters of cells, typically found in fluid specimens, to screen for or diagnose various conditions, including cancer. It represents a minimally invasive procedure offering rapid results and finds wide application across diverse anatomical regions. Cytological tests include scraping or brushing tissue surfaces, collecting body fluids, fine needle aspirations, and other tissue biopsies. Examples of cytology, such as exfoliative and intervention cytology, are employed depending on the sampling method [18,19].

In contrast, histopathology centres on examining tissues obtained through biopsy or surgical excision of organs. It involves scrutinizing tissue structures under a microscope to assess cell architecture and cell arrangement and discern disease features. Histopathology is critical in diagnosing various illnesses, particularly cancer, and is a cornerstone in disease monitoring, treatment evaluation, and medical knowledge advancement. The histopathological process encompasses sample processing, sectioning into thin slices, staining, and microscopic examination to delineate tissue characteristics. Performed by pathologists, histopathology significantly contributes to disease management and treatment advancements [20,21].

Tumour Markers (CEA and CA-125)

Carcinoembryonic antigen (CEA) is a tumour marker widely employed in clinical practice for various solid malignant carcinomas spanning lung, oesophagal, colorectal, and epithelial ovarian cancers [22]. Although CEA proves valuable in diagnosing gynaecological malignant tumours and breast cancer, its diagnostic sensitivity or specificity alone may not suffice for diagnosing epithelial ovarian cancer [22]. Augmenting CEA with other tumour markers, such as cancer antigen 125 (CA-125) and cancer antigen 19-9 (CA 19-9), can bolster diagnostic accuracy, furnishing a more comprehensive evaluation for epithelial ovarian cancer [22]. CA-125, alternatively known as mucin 16 or MUC16, denotes a protein encoded by the MUC16 gene and exhibits elevated levels in specific cancer types, notably ovarian cancer [22,23]. While heightened CA-125 levels commonly correlate with ovarian cancer, they can also manifest in individuals devoid of ovarian malignancy, thereby diminishing diagnostic specificity [22,23]. Primarily utilized to monitor ovarian cancer treatment efficacy, elevated CA-125 levels may manifest in uterine, cervical, pancreatic, hepatic, colonic, mammary, pulmonary, and gastrointestinal cancers and specific noncancerous conditions [22,23].

Staging and prognostic factors

Classification Systems (Sugarbaker and Ronnett)

The classification systems proposed by Sugarbaker and Ronnett are pivotal in categorizing PMP based on histological characteristics, thereby informing treatment decisions and prognostic assessments. Sugarbaker's classification stratifies PMP into low-grade and high-grade categories, with survival outcomes correlating closely with these grades [4]. Conversely, Ronnett et al. initially delineated PMP into two groups, underscoring the significance of histological grading in prognostication and determining potential adjuvant therapy options [10]. These classification frameworks assume critical importance in stratifying PMP cases according to disease aggressiveness, thereby assisting clinicians in devising appropriate management strategies, including the extent of surgical intervention and the potential necessity for adjuvant therapies. By segregating PMP into discrete groups based on histological features, these systems facilitate the prediction of patient outcomes and customize treatment plans to optimize patient care and prognosis [24].

Factors Influencing Prognosis

Several factors influence the prognosis of PMP, each playing a crucial role in determining the likelihood of treatment success and disease recurrence. Firstly, the extent and location of the tumour significantly impact surgical outcomes and recurrence rates. Smaller tumours confined to the appendix generally carry a more favourable prognosis than larger ones that have disseminated throughout the peritoneal cavity [25]. Moreover, the histological grade of the tumour, ranging from well-differentiated (low-grade) to poorly differentiated (high-grade), is a crucial determinant of prognosis. Low-grade tumours typically portend a better prognosis than high-grade tumours [25,26]. Additionally, the patient's age and overall health status are paramount considerations. Older patients and those with underlying medical conditions may face a poorer prognosis due to heightened susceptibility to treatment complications [25].

The success of surgical and chemotherapy treatments also significantly influences prognosis. Complete tumour removal through CRS and successful HIPEC administration is associated with improved prognostic outcomes [25,26]. Furthermore, the presence of coexisting medical conditions such as diabetes or heart disease can impact overall health and treatment tolerance, thus influencing prognosis [25]. Additionally, the patient's response to treatment serves as a pivotal prognostic indicator. Favourable treatment responses and absence of disease post-treatment indicate a better prognosis, whereas disease recurrence or progression signals a poorer prognosis [25]. These factors collectively underscore the challenge of predicting prognosis for PMP patients, with survival rates varying based on individual circumstances. Nonetheless, with appropriate treatment and comprehensive management strategies, patients can achieve enhanced quality of life and long-term survival outcomes [25,26].

Predictive Models and Scoring Systems

A novel nomogram prediction model has been developed, integrating quantitative analysis of pathological images (hematoxylin & eosin stained) to forecast the prognosis of PMP patients [27]. This model aims to enhance prognostic prediction beyond traditional clinicopathological factors. Additionally, a preoperative CT-based scoring system has been proposed to anticipate the resectability of PMP before surgery [28]. This initiative could guide treatment planning and patient selection for CRS. Moreover, pathological prognostic factors, such as tumour grade and disease extent, have emerged as pivotal predictors of outcomes in PMP [29]. These factors are integrated into staging systems like the American Joint Committee on Cancer (AJCC) staging for appendiceal carcinomas. Furthermore, preoperative nutritional status, assessed through indicators such as weight loss, red blood cell count, and albumin levels, has demonstrated its impact on prognosis in PMP patients [30]. A modified Glasgow Prognostic Score incorporating these parameters may prove beneficial. Furthermore, prior surgical history, quantified by a peritoneal surface segmentation (PSS) score, has been identified as an independent predictor of overall survival following CRS and HIPEC [29,30]. Patients with a history of more extensive prior surgeries tend to experience poorer outcomes.

Implications for Treatment Planning

Aggressive surgical debulking, commonly known as CRS, stands as the cornerstone of treatment for PMP, often complemented by HIPEC [2,31]. The primary objective is to eradicate all discernible tumour deposits within the peritoneal cavity, aiming for complete removal. The underlying pathology and extent of disease dissemination play pivotal roles in determining prognosis and directing treatment strategies. PMP arising from low-grade appendiceal mucinous neoplasms typically portends a more favourable prognosis than high-grade disseminated neoplasms [2,31]. Careful patient evaluation is imperative to ascertain eligibility for the intensive CRS and HIPEC procedure, as it necessitates robust overall health and the capacity to excise all visible diseases without compromising vital organs [2,31]. For patients deemed ineligible for CRS and HIPEC, alternative treatment modalities include debulking surgery, systemic chemotherapy, and palliative care to alleviate symptoms [2,31]. Specialized multidisciplinary centres equipped with expertise in PMP management are recommended for optimal treatment delivery, given the rarity and complexity of the condition [2,31]. Ongoing research endeavours are directed towards refining treatment approaches, encompassing the exploration of novel chemotherapeutic agents and investigating the potential role of early postoperative intraperitoneal chemotherapy [2,31].

Quality of life and survivorship

Impact of PMP and Its Treatments on Quality of Life

Research on PMP and its therapeutic interventions has demonstrated a notable influence on the quality of life of affected individuals. Investigations have underscored that the health-related quality of life remains excellent and enduring after CRS and intraperitoneal chemotherapy for PMP, with improvements in emotional well-being despite potential short-term recovery complications [3,4]. Preoperatively, patients with PMP may encounter impairments in emotional and social functional domains; however, postoperative outcomes reveal an enhancement in these aspects, indicative of a positive treatment impact on quality of life [32]. Additionally, findings from a prospective longitudinal study highlight that most patients return to preoperative levels of functioning within three to six months post surgery, with some maintaining survival without significant deterioration in the quality of life up to one year post treatment [3]. This underscores that despite the challenges associated with PMP and its therapeutic modalities, patients stand to experience favourable outcomes in terms of emotional well-being and functional recovery, ultimately contributing to an acceptable quality of life [32,33].

Rehabilitation and Supportive Care Services

Rehabilitation and supportive care services are essential in treating patients grappling with PMP. These services are geared towards augmenting the quality of life, physical functionality, and emotional resilience of individuals undergoing treatment for PMP. Patients stand to benefit from a spectrum of supportive care interventions, including psychological assistance, nutritional guidance, pain management, and physical therapy, all aimed at facilitating post-surgical recovery [31]. Tailored rehabilitation programs, designed to meet the unique needs of each patient, serve to optimize recovery and functional outcomes after CRS and HIPEC for PMP. These programs may zero in on restoring mobility, enhancing strength, and fostering overall physical functionality, all integral facets of the recuperative journey [34]. Moreover, psychological support assumes paramount importance for patients grappling with the multifaceted challenges associated with PMP treatment. Counselling services and support groups offer a nurturing environment wherein patients can find solace, navigate the intricacies of their diagnosis and treatment, and fortify their overall well-being throughout the treatment trajectory [35].

Patient Perspectives and Advocacy

Patient perspectives and advocacy are pivotal in managing PMP. Individuals grappling with PMP encounter a journey fraught with complexity and challenges, necessitating not only medical expertise but also emotional sustenance and empowerment. The research underscores the indispensability of patient-reported outcomes and quality-of-life assessments in comprehending the impact of PMP treatment on affected individuals [33,36]. Advocacy groups and patient support networks emerge as invaluable resources, furnishing information, emotional sustenance, and vital support to individuals and families grappling with PMP. These entities play instrumental roles in heightening awareness about the condition, facilitating access to specialized care, and advocating for enhanced treatment options and research funding [37]. Moreover, patient perspectives wield considerable influence in shaping healthcare policies, research agendas, and treatment protocols. By articulating their experiences, grappling with challenges, and elucidating their needs, patients contribute substantially to formulating more patient-centric care paradigms, thus elevating the overall quality of care rendered to individuals with PMP [37].

Future directions and research needs

Molecular Characterization and Targeted Therapy

Investigating the molecular underpinnings of PMP is pivotal for pinpointing potential therapeutic targets. This entails delving into genetic mutations, such as RAS mutations, and advancing the development of targeted therapies like cetuximab, renowned for its efficacy against cancers boasting active epidermal growth factor receptors (EGFR) [38]. By elucidating the molecular landscape of PMP, researchers can identify specific vulnerabilities within cancer cells, paving the way for developing tailored treatment approaches that directly target these aberrations, potentially enhancing treatment efficacy and patient outcomes.

Improved Diagnostic Techniques

Advancements in diagnostic methodologies are imperative to detect PMP at earlier stages, facilitating more prompt and efficacious treatment interventions and potentially improving patient prognoses. This imperative underscores the need for refining biomarkers or imaging techniques capable of accurately detecting the disease, even in its incipient stages [39]. Enhanced diagnostic capabilities empower clinicians to initiate treatment interventions promptly before the disease progresses to advanced stages, optimizing therapeutic efficacy and patient outcomes.

Enhanced Understanding of Pathophysiology

Further exploring the pathophysiological mechanisms underpinning PMP is imperative to glean comprehensive insights into the disease's aetiology and progression. A deeper understanding of the biological mechanisms driving PMP enables researchers to devise more targeted and effective treatment strategies, potentially yielding improved patient outcomes [40]. By unravelling the intricacies of PMP pathophysiology, researchers can identify novel therapeutic targets and develop interventions that specifically target disease-driving pathways, thus potentially offering new avenues for therapeutic intervention and enhancing patient prognoses.

Development of New Treatment Strategies

While CRS, followed by HIPEC, represents the current standard of care for PMP, there is a pressing need to explore and develop alternative or complementary treatment modalities. These novel therapeutic strategies aim to augment existing treatment protocols, improving patient outcomes and quality of life [34]. By diversifying the therapeutic armamentarium, researchers aim to address the varied needs of PMP patients, offering personalized treatment approaches tailored to individual disease characteristics and patient preferences.

Personalized Medicine

The advent of personalized medicine approaches, such as precision medicine and immunotherapy, holds promise for revolutionizing treatment outcomes in PMP. Research endeavours in these domains seek to harness patients' unique genetic profiles and disease characteristics to tailor treatment regimens to individual patient's optimal needs [41]. By leveraging personalized medicine approaches, clinicians aim to optimize treatment efficacy while minimizing adverse effects, ultimately improving patient outcomes and enhancing overall treatment experiences.

Improved Patient Selection for Surgery

The judicious selection of patients for surgical intervention constitutes a pivotal aspect of managing PMP. By delving into the factors predictive of successful surgical outcomes and refining preoperative evaluation tools, researchers strive to enhance patient selection protocols, ultimately optimizing treatment outcomes [42]. Through meticulous patient selection, clinicians can identify individuals most likely to benefit from surgical intervention while minimizing the risk of postoperative complications, thus improving overall treatment efficacy and patient satisfaction.

Enhanced Supportive Care

The impact of PMP and its treatment on patients' quality of life underscores the critical need for more effective supportive care strategies. Investigations into advanced pain management techniques, nutritional support interventions, and psychosocial support initiatives aim to ameliorate the adverse effects of PMP and its treatment modalities, thereby enhancing patient well-being and treatment adherence [43]. By bolstering supportive care measures, clinicians strive to mitigate treatment-related side effects, optimize patient comfort, and foster improved treatment compliance, ultimately leading to enhanced treatment outcomes and overall patient satisfaction.

Global Collaboration and Standardization

Establishing international consensus statements and guidelines for PMP diagnosis and management has significantly elevated the standard of care globally. Sustained collaboration and standardization efforts are imperative to ensure the widespread adoption of best practices across healthcare settings [36]. By fostering global collaboration and standardization, clinicians can harmonize diagnostic and treatment protocols, optimize resource allocation, and improve treatment consistency, enhancing overall treatment efficacy and patient outcomes globally.

Investigation of Novel Therapies

The exploration of innovative therapeutic modalities, including immunotherapy and targeted therapies, holds promise for revolutionizing treatment outcomes in PMP. Research endeavours in these domains aim to uncover novel therapeutic targets and develop tailored treatment regimens that exploit the unique biological characteristics of PMP [44]. By delving into novel therapeutic avenues, researchers strive to expand the treatment armamentarium, offering patients cutting-edge treatment options that may yield improved treatment responses and enhanced long-term outcomes.

Investigation of the Role of RAS Mutations

Despite advances in our understanding of PMP, the precise role of RAS mutations in disease pathogenesis still needs to be understood. Further research into the impact of RAS mutations on disease progression and treatment responses is imperative to elucidate their significance and inform the development of targeted therapeutic interventions [45]. By unravelling the intricacies of RAS mutations in PMP, researchers aim to identify novel therapeutic targets and refine treatment strategies, ultimately enhancing treatment efficacy and patient outcomes.

## Conclusions

In conclusion, this comprehensive review has shed light on various aspects of PMP, ranging from its definition and historical context to its epidemiology, diagnosis, management, and prognosis. PMP, characterized by the accumulation of mucinous ascites within the peritoneal cavity, poses significant diagnostic and therapeutic challenges due to its rarity and complex clinical presentation. However, advancements in diagnostic imaging, molecular biology, and treatment strategies, particularly CRS combined with HIPEC, have revolutionized PMP management and improved patient outcomes. Nevertheless, challenges such as disease recurrence, treatment-related morbidity, and the need for long-term surveillance persist, underscoring the importance of a multidisciplinary approach and specialized care for PMP patients. Looking ahead, ongoing research efforts hold promise for further refining treatment algorithms, identifying novel therapeutic targets, and ultimately improving the prognosis and quality of life for individuals affected by PMP. With continued collaboration among clinicians, researchers, and patient advocates, the outlook for PMP patients is expected to become increasingly optimistic, offering hope for better outcomes and enhanced survivorship in the years to come.
